# Risk factors and intervention strategies for lower extremity deep vein thrombosis after intravenous thrombolysis for acute ischemic stroke

**DOI:** 10.3389/fcvm.2026.1832515

**Published:** 2026-07-07

**Authors:** Xiaomeng Zhang, Wenjuan Geng, Liang Wei, Yan Li

**Affiliations:** 1 Department of Neurology, People's Hospital of Huantai County, Zibo, China; 2 Department of Medical Affairs, People's Hospital of Huantai County, Zibo, China

**Keywords:** acute ischemic stroke, deep vein thrombosis, intravenous thrombolysis, prevention gap, retrospective cohort, thromboprophylaxis

## Abstract

**Objective:**

This study examined the association between absence of early pharmacological thromboprophylaxis and in-hospital DVT after IVT for AIS, and further explored the clinical characteristics associated with failure to initiate timely prophylaxis.

**Methods:**

We conducted a single-center retrospective cohort study of 197 patients with AIS treated with IVT between January 2021 and April 2024. Early pharmacological thromboprophylaxis was defined as anticoagulant prophylaxis initiated within 24–48 h after IVT after repeat neuroimaging excluded intracranial hemorrhage. The primary outcome was in-hospital lower extremity DVT confirmed by venous ultrasonography. Multivariable logistic regression was used to identify factors associated with non-initiation of early prophylaxis and to examine the association between absence of early prophylaxis and DVT.

**Results:**

Among 197 patients, 118 (59.9%) received early prophylaxis and 79 (40.1%) did not. Overall, 32 patients (16.2%) developed in-hospital DVT. DVT occurred more frequently in patients without early prophylaxis than in those receiving early prophylaxis (30.4% vs. 6.8%, *P* < 0.001). After adjustment, absence of early prophylaxis was associated with higher odds of DVT (adjusted OR 3.16, 95% CI 1.19–8.42, *P* = 0.021). Other factors retained in the adjusted model included atrial fibrillation, higher NIHSS score, prolonged bed rest, and elevated D-dimer. Patients with atrial fibrillation, greater stroke severity, and higher D-dimer levels were also less likely to receive early prophylaxis.

**Conclusions:**

Among patients with AIS treated with IVT, absence of early pharmacological thromboprophylaxis was associated with higher odds of in-hospital DVT. Notably, a risk-treatment paradox was observed: patients with higher baseline thrombotic risk (e.g., severe stroke, atrial fibrillation, elevated D-dimer) were less likely to receive early prophylaxis, presumably due to concerns about post-thrombolysis hemorrhagic transformation. These findings suggest a clinically relevant prevention gap, although the observed association should be interpreted cautiously given the retrospective design and non-randomized treatment allocation.

## 1 Introduction

Acute ischemic stroke (ACI) remains a leading cause of mortality and long-term disability worldwide, imposing a substantial global disease burden. Data from the Global Burden of Disease (GBD) study indicate that cerebral infarction is among the foremost contributors to years of healthy life lost globally (1). The situation is particularly severe in China, where epidemiological studies have shown that the mortality rate of cerebral infarction is approximately four times higher than that reported in Western countries. In 2020 alone, an estimated 3.4 million new cases of ACI occurred among individuals aged 40 years and older in China, with approximately 2.3 million related deaths, posing a major challenge to both the public health system and socioeconomic development (2). Consequently, identifying effective therapeutic strategies and improving clinical outcomes remain key research priorities in contemporary neuroscience.

Intravenous thrombolysis (IVT), particularly with recombinant tissue plasminogen activator (rt-PA), is currently the most effective pharmacological treatment for acute ischemic stroke in the hyperacute phase and has been shown to significantly reduce post-stroke disability (3, 4). However, patients with ACI are inherently at high risk for venous thromboembolism (VTE)—especially lower extremity deep vein thrombosis (LEDVT)—due to neurological deficits, limb paralysis, reduced mobility, and prolonged bed rest (5). VTE encompasses both deep vein thrombosis and its most severe complication, pulmonary embolism (PE), which is potentially fatal (6). Previous studies have reported that the mortality rate within one month after VTE diagnosis can reach approximately 12% (5, 7). Therefore, once ACI patients develop LEDVT, they may experience aggravated limb swelling and pain, delayed neurological recovery, and, more critically, a markedly increased risk of life-threatening PE. These complications substantially diminish the survival benefits conferred by IVT and impose a significant burden on patients, their families, and society.

To date, numerous studies have independently examined the risk factors for ACI—such as hypertension, diabetes mellitus, atrial fibrillation, hyperhomocysteinemia, and inflammatory markers (8–10)—as well as the established risk factors for LEDVT, including advanced age, immobilization, malignancy, hypercoagulable states, and a history of thrombosis (11–13). However, studies that integrate these two conditions, particularly those focusing on ACI patients receiving IVT, remain limited. Intravenous thrombolysis activates the fibrinolytic system to dissolve thrombi and may theoretically alter the systemic coagulation–fibrinolysis balance. At the same time, the increased risk of hemorrhagic complications associated with IVT often restricts the early use of pharmacological anticoagulation for VTE prevention, rendering thrombotic risk management in this population particularly complex and challenging (14, 15).

Current guidelines recommend that, in high-risk stroke patients, prophylactic low-molecular-weight heparin or fondaparinux be initiated 24–48 h after thrombolysis, once intracranial hemorrhage is excluded (14, 15). In clinical practice, however, adherence to this recommendation is highly variable, and many patients may not receive timely pharmacological prophylaxis due to concerns about bleeding, the severity of neurological deficits, or other clinical factors. This “prevention gap”—the failure to implement early pharmacological prophylaxis despite guideline recommendations—may contribute to an increased risk of in-hospital DVT. While several studies have developed risk prediction models or nomograms for post-stroke DVT (11, 13, 16), few have specifically examined the association between the absence of early prophylaxis and DVT occurrence in the IVT population, nor have they characterized which patients are most likely to experience this prevention gap.

Against this background, the present retrospective cohort study was designed to examine whether the absence of early pharmacological thromboprophylaxis after IVT was associated with in-hospital DVT in patients with AIS, and to describe the clinical characteristics linked to non-initiation of timely prophylaxis. Given the non-randomized nature of prophylaxis decisions in routine practice, we regarded early prophylaxis status primarily as a treatment-practice exposure that may also reflect underlying stroke severity, bleeding concerns, and physician judgment.

## 2 Materials and methods

### 2.1 Study design and population

This single-center retrospective cohort study consecutively enrolled patients with AIS who received IVT at the Department of Neurology, People's Hospital of Huantai County, between January 2021 and April 2024. AIS was diagnosed according to the Chinese Guidelines for the Diagnosis and Treatment of Acute Ischemic Stroke (2018) and confirmed by cranial computed tomography (CT) or magnetic resonance imaging (MRI) demonstrating new infarct lesions corresponding to neurological deficits. All patients received intravenous recombinant tissue plasminogen activator (rt-PA, alteplase) or urokinase within 4.5 h of symptom onset, following standard eligibility criteria for thrombolysis.

### 2.2 Inclusion and exclusion criteria

Inclusion criteria were: (1) age ≥18 years; (2) confirmed diagnosis of AIS and receipt of IVT; (3) admission cranial CT ruling out intracranial hemorrhage or extensive early infarct signs; (4) completion of at least one lower extremity venous ultrasound examination during hospitalization; (5) complete clinical records covering all required variables for this study.

Exclusion criteria were: (1) absolute or relative contraindications to thrombolysis (e.g., active internal bleeding, recent intracranial surgery, known history of intracranial hemorrhage, uncontrolled severe hypertension); (2) symptomatic intracranial hemorrhage or rapid clinical deterioration post-thrombolysis with anticipated short survival; (3) pre-existing lower extremity DVT or pulmonary embolism on admission; (4) severe hepatic or renal dysfunction, hematological disorders, or terminal malignancy with life expectancy <3 months; (5) incomplete clinical data precluding valid analysis.

Patients with known hereditary or acquired thrombophilia (e.g., antiphospholipid syndrome, protein C or S deficiency, antithrombin deficiency) were not systematically excluded, as routine thrombophilia screening was not performed in this cohort. No patient had a documented diagnosis of such conditions in the medical records.

### 2.3 Data collection and variable definitions

Data were retrospectively extracted from the hospital electronic medical record system (HIS) and the picture archiving and communication system (PACS) using a standardized data collection form. Two trained researchers independently extracted and cross-verified all data; discrepancies were resolved by a third investigator.

The following variables were collected:

Demographic characteristics: age, sex.

Medical history and lifestyle: hypertension, diabetes mellitus, coronary artery disease, atrial fibrillation, prior stroke, prior DVT, malignancy, smoking, alcohol consumption.

Clinical characteristics: systolic and diastolic blood pressure at admission; National Institutes of Health Stroke Scale (NIHSS) scores assessed before thrombolysis and 24 h post-thrombolysis; presence of lower limb paralysis before thrombolysis (defined as muscle strength ≤ grade 3 in the affected lower extremity); duration of absolute bed rest (>3 days defined as prolonged bed rest); use of intermittent pneumatic compression (IPC) during hospitalization.

Thrombolysis parameters: onset-to-needle time (minutes), type of thrombolytic agent (alteplase or urokinase).

Laboratory parameters: initial laboratory values obtained on admission, including D-dimer (mg/L), fibrinogen (g/L), low-density lipoprotein cholesterol (LDL-C, mmol/L), homocysteine (μmol/L), high-sensitivity C-reactive protein (hs-CRP, mg/L), and glycated hemoglobin (HbA1c, %).

Outcome variables: in-hospital DVT (site, type, and timing), bleeding events (type, severity, timing).

In our institution, no standardized risk scoring system (e.g., Caprini or Padua score) was routinely used to guide pharmacological thromboprophylaxis decisions during the study period. The decision to initiate early prophylaxis was made by the treating clinician based on clinical judgment, including stroke severity (NIHSS), immobility duration, atrial fibrillation status, D-dimer level, and perceived bleeding risk. This absence of a structured risk assessment tool likely contributed to the observed variability in prophylaxis allocation.

### 2.4 Key definitions

Early pharmacological thromboprophylaxis was defined as follows: Prophylactic-dose enoxaparin (40 mg once daily, reduced to 30 mg if eGFR <30 mL/min) or fondaparinux (2.5 mg once daily) was initiated within 24–48 h after IVT. Repeat brain imaging (CT or MRI) had to exclude intracranial hemorrhage or significant hemorrhagic transformation before initiation. Patients who did not receive any pharmacological prophylaxis during the first 48 h post-thrombolysis were classified as the “no early prophylaxis” group. In routine practice, the decision to initiate prophylaxis within this time window was made by the treating clinical team after repeat neuroimaging and overall bleeding-risk assessment, rather than by a predefined study protocol. Criteria for not initiating early prophylaxis: No formal criteria were used; decisions were based on clinician judgment. Reasons inferred from notes included repeat imaging showing hemorrhage, large infarct size, concurrent antiplatelet use, or patient refusal, but these were not systematically recorded.

Prolonged absolute bed rest (>3 days) was defined as medically mandated strict bed rest lasting more than 72 h due to neurological deficits (e.g., lower limb paralysis, impaired consciousness) or other clinical conditions limiting mobility.

Lower limb paralysis was defined as muscle strength ≤ grade 3 on the Medical Research Council (MRC) scale for the affected lower extremity, assessed before thrombolysis.

DVT was diagnosed according to the Guidelines for the Diagnosis and Treatment of Deep Vein Thrombosis (3rd Edition) and confirmed by lower extremity color Doppler ultrasonography. Ultrasound examinations were performed either triggered by clinical symptoms (limb swelling, pain, or tenderness) or as routine screening for high-risk patients (typically on days 3–5 post-admission). DVT was categorized by location (left lower limb, right lower limb, bilateral) and type (proximal, involving popliteal vein or above; distal, confined to calf veins). Time from thrombolysis to DVT diagnosis was recorded in days. Because ultrasonography was performed according to routine clinical practice rather than a uniform study-mandated surveillance schedule, outcome detection may have varied according to symptom burden, immobilization status, and clinician judgment.

Bleeding events were classified according to International Society on Thrombosis and Haemostasis (ISTH) criteria. Major bleeding was defined as: (1) symptomatic ICH; (2) bleeding associated with a hemoglobin drop of ≥2 g/dL; (3) bleeding requiring transfusion of ≥2 units of packed red blood cells; (4) bleeding occurring in a critical site (e.g., retroperitoneal, intra-articular, pericardial). All bleeding events were confirmed by imaging (CT for ICH, endoscopy for gastrointestinal bleeding) or clinical documentation.

### 2.5 Statistical analysis

Statistical analyses were performed using SPSS version 26.0 (IBM Corp., Armonk, NY, USA) and R software version 4.1.0 (R Foundation for Statistical Computing, Vienna, Austria). Continuous variables were described as mean ± standard deviation or median (interquartile range), depending on their distribution, and categorical variables were expressed as frequencies and percentages. Between-group comparisons were performed using the Student's *t* test or Mann–Whitney *U*-test for continuous variables, and the chi-square test or Fisher's exact test for categorical variables, as appropriate.

The main analysis focused on two prespecified multivariable logistic regression models. Model A evaluated factors associated with the absence of early pharmacological thromboprophylaxis. Model B evaluated the association between absence of early prophylaxis and in-hospital DVT. Variables entered into multivariable analyses were selected based on clinical plausibility and baseline comparisons, while keeping the number of covariates parsimonious in relation to the number of outcome events.

Results are presented as adjusted odds ratios (ORs) with 95% confidence intervals (CIs). Given the retrospective observational design and the non-randomized nature of prophylaxis decisions in routine practice, these estimates were interpreted as associations rather than causal effects.

Three sensitivity analyses were prespecified to test the consistency of the main findings. First, D-dimer was modeled as a log-transformed continuous variable instead of a categorized variable. Second, patients with extremely short or prolonged lengths of hospital stay were excluded. Third, the propensity score for receipt of early prophylaxis was additionally included in the adjusted model. The propensity score was estimated using logistic regression based on baseline characteristics considered clinically relevant to prophylaxis allocation.

Subgroup analyses and cumulative risk-stratified analyses were treated as exploratory and supportive only. In view of the single-center retrospective design, modest sample size, and limited number of DVT events, these additional analyses were interpreted cautiously and were not considered primary evidence. All statistical tests were two-sided, and statistical significance was defined as *P* < 0.05.

## 3 Results

### 3.1 Study population and prophylaxis patterns

**Figure 1:**
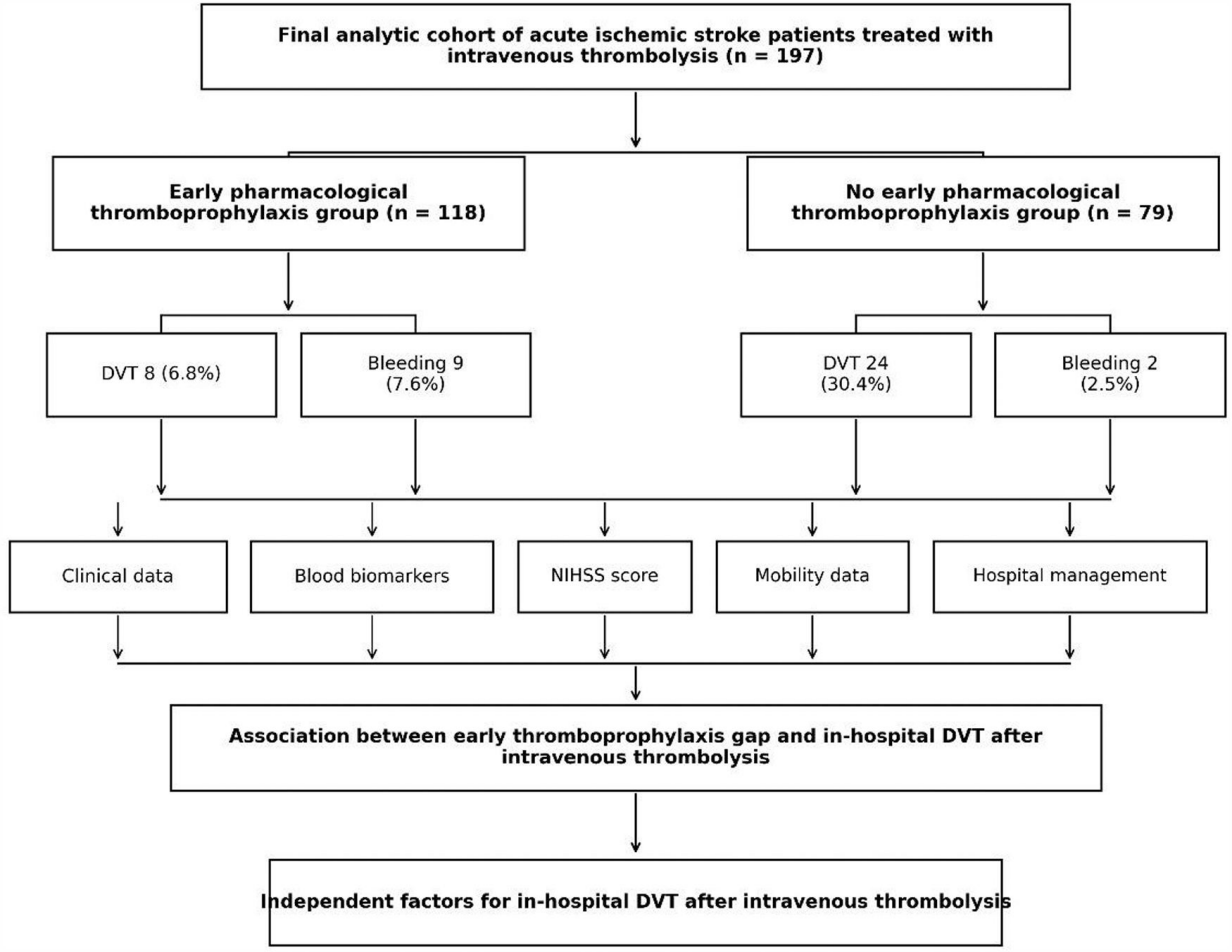
Study flowchart.

A total of 197 patients with AIS who received IVT between January 2021 and April 2024 were included in the final analysis. Among them, 118 patients (59.9%) received early pharmacological thromboprophylaxis within 24–48 h after IVT following repeat neuroimaging that excluded intracranial hemorrhage, whereas 79 patients (40.1%) did not receive pharmacological prophylaxis during the first 48 h after thrombolysis. Overall, in-hospital lower extremity DVT occurred in 32 patients (16.2%), and bleeding events were recorded in 11 patients (5.6%), including 4 cases (2.0%) of symptomatic intracranial hemorrhage and 7 cases (3.6%) of major extracranial bleeding. The patient screening and group allocation process is presented in Figure 1.

### 3.2 Baseline characteristics according to early pharmacological prophylaxis

Baseline clinical characteristics stratified by receipt of early pharmacological thromboprophylaxis are summarized in Table 1. Compared with patients who received early prophylaxis, those without early prophylaxis were older and more likely to have atrial fibrillation. They also had more severe neurological impairment, reflected by higher pre-thrombolysis NIHSS scores and a greater proportion of NIHSS ≥10, as well as higher frequencies of lower limb paralysis and prolonged absolute bed rest for more than 3 days. In addition, the no-early-prophylaxis group showed higher levels of D-dimer, fibrinogen, homocysteine, and hs-CRP, and was less likely to receive IPC. Importantly, the in-hospital DVT rate was substantially higher in the no-early-prophylaxis group than in the early prophylaxis group (30.4% vs. 6.8%, *P* < 0.001). By contrast, no significant between-group differences were observed in sex, hypertension, diabetes mellitus, coronary artery disease, prior stroke, malignancy, smoking, alcohol consumption, onset-to-needle time, or thrombolytic agent use. Detailed baseline imbalance and post-adjustment balance diagnostics are shown in Supplementary Table S1.

**Table 1:** Baseline characteristics of AIS patients stratified by receipt of early pharmacological thromboprophylaxis.

Variable	Total (*N* = 197)	Early prophylaxis (*n* = 118)	No early prophylaxis (*n* = 79)	*P* value
Demographics				
Age (years), mean ± SD	69.1 ± 12.3	66.8 ± 12.1	72.5 ± 11.8	0.001
Age ≥75 years, *n* (%)	75 (38.1)	36 (30.5)	39 (49.4)	0.007
Male sex, *n* (%)	122 (61.9)	75 (63.6)	47 (59.5)	0.565
Medical history, *n* (%)				
Hypertension	142 (72.1)	85 (72.0)	57 (72.2)	0.985
Diabetes mellitus	79 (40.1)	47 (39.8)	32 (40.5)	0.924
Coronary artery disease	56 (28.4)	31 (26.3)	25 (31.6)	0.412
Atrial fibrillation	63 (32.0)	26 (22.0)	37 (46.8)	<0.001
Prior stroke	61 (31.0)	35 (29.7)	26 (32.9)	0.629
Prior DVT	7 (3.6)	3 (2.5)	4 (5.1)	0.438
Malignancy	14 (7.1)	7 (5.9)	7 (8.9)	0.427
Smoking	58 (29.4)	37 (31.4)	21 (26.6)	0.467
Alcohol	40 (20.3)	26 (22.0)	14 (17.7)	0.458
Clinical characteristics				
Pre-thrombolysis NIHSS, median (Q1, Q3)	6 (3, 11)	4 (2, 8)	12 (7, 18)	<0.001
NIHSS ≥10, *n* (%)	64 (32.5)	21 (17.8)	43 (54.4)	<0.001
Lower limb paralysis, *n* (%)	90 (45.7)	42 (35.6)	48 (60.8)	<0.001
Absolute bed rest >3 days, *n* (%)	65 (33.0)	25 (21.2)	40 (50.6)	<0.001
Use of IPC, *n* (%)	82 (41.6)	58 (49.2)	24 (30.4)	0.009
Thrombolysis parameters				
Onset-to-needle time (min), mean ± SD	179 ± 47	176 ± 45	183 ± 50	0.305
Alteplase use, *n* (%)	160 (81.2)	98 (83.1)	62 (78.5)	0.424
Laboratory parameters				
D-dimer (mg/L), mean ± SD	1.0 ± 1.1	0.7 ± 0.6	1.5 ± 1.4	<0.001
D-dimer ≥2.0 mg/L, *n* (%)	36 (18.3)	8 (6.8)	28 (35.4)	<0.001
Fibrinogen (g/L), mean ± SD	3.6 ± 1.1	3.4 ± 1.0	3.9 ± 1.2	0.002
LDL-C (mmol/L), mean ± SD	2.6 ± 0.8	2.6 ± 0.8	2.7 ± 0.9	0.398
Homocysteine (μmol/L), median (Q1, Q3)	15.6 (11.5, 21.2)	14.8 (11.0, 20.1)	17.2 (12.5, 24.0)	0.048
Hs-CRP (mg/L), median (Q1, Q3)	5.6 (2.3, 11.4)	4.2 (1.9, 9.5)	8.1 (3.5, 16.2)	0.015
Outcomes				
In-hospital DVT, *n* (%)	32 (16.2)	8 (6.8)	24 (30.4)	<0.001
Any bleeding event, *n* (%)	11 (5.6)	9 (7.6)	2 (2.5)	0.123
Symptomatic ICH	4 (2.0)	3 (2.5)	1 (1.3)	0.654
Major extracranial bleeding	7 (3.6)	6 (5.1)	1 (1.3)	0.248
Hospital stay (days), median (Q1, Q3)	12 (8, 18)	10 (7, 15)	15 (10, 22)	0.002

SD, standard deviation; Q1, first quartile; Q3, third quartile; NIHSS, National Institutes of Health Stroke Scale; IPC, intermittent pneumatic compression; LDL-C, low-density lipoprotein cholesterol; hs-CRP, high-sensitivity C-reactive protein; DVT, deep vein thrombosis; ICH, intracranial hemorrhage.

### 3.3 Factors associated with absence of early pharmacological prophylaxis

To identify clinical factors independently associated with failure to initiate early pharmacological thromboprophylaxis, we performed multivariable logistic regression analysis (Table 2, Model A). Higher pre-thrombolysis NIHSS score was independently associated with an increased likelihood of not receiving early prophylaxis (OR = 1.15 per point, 95% CI: 1.07–1.23, *P* < 0.001). Atrial fibrillation (OR = 2.98, 95% CI: 1.45–6.12, *P* = 0.003) and D-dimer ≥2.0 mg/L (OR = 4.23, 95% CI: 1.58–11.32, *P* = 0.004) were also independent predictors of a prophylaxis gap. Age showed a borderline association with omission of early prophylaxis (OR = 1.25 per 10-year increase, 95% CI: 0.98–1.60, *P* = 0.072). These findings suggest that patients perceived as clinically more severe or potentially more complex were less likely to receive guideline-recommended early prophylaxis.

**Table 2:** Multivariable logistic regression models for absence of early prophylaxis (model A) and in-hospital DVT (model B).

Variable	Model A (Outcome: No early prophylaxis)		Model B (Outcome: In-hospital DVT)	
	Adjusted OR (95% CI)	*P*	Adjusted OR (95% CI)	*P*
Absence of early prophylaxis (vs. received)	–	–	3.16 (1.19–8.42)	0.021
Age (per 10-year increase)	1.25 (0.98–1.60)	0.072	1.08 (0.75–1.56)	0.683
Atrial fibrillation	2.98 (1.45–6.12)	0.003	3.39 (1.22–9.41)	0.019
Pre-thrombolysis NIHSS (per 1-point)	1.15 (1.07–1.23)	<0.001	1.20 (1.07–1.35)	0.003
Absolute bed rest >3 days	1.88 (0.88–4.02)	0.102	7.46 (2.40–23.21)	<0.001
D-dimer ≥2.0 mg/L	4.23 (1.58–11.32)	0.004	5.21 (1.78–15.28)	0.003
Fibrinogen (per 1 g/L)	1.12 (0.82–1.52)	0.48	1.08 (0.68–1.70)	0.751
hs-CRP (per 1 mg/L)	1.02 (0.98–1.06)	0.322	1.03 (0.98–1.09)	0.228
Use of IPC	0.68 (0.35–1.32)	0.251	0.61 (0.22–1.70)	0.344

Model A includes all variables with *P* < 0.10 in Table 1 comparison between prophylaxis groups. Model B includes the same covariates plus the exposure of interest (absence of early prophylaxis). ORs are adjusted for all variables listed. IPC, intermittent pneumatic compression.

### 3.4 Association between absence of early prophylaxis and in-hospital DVT

**Figure 2:**
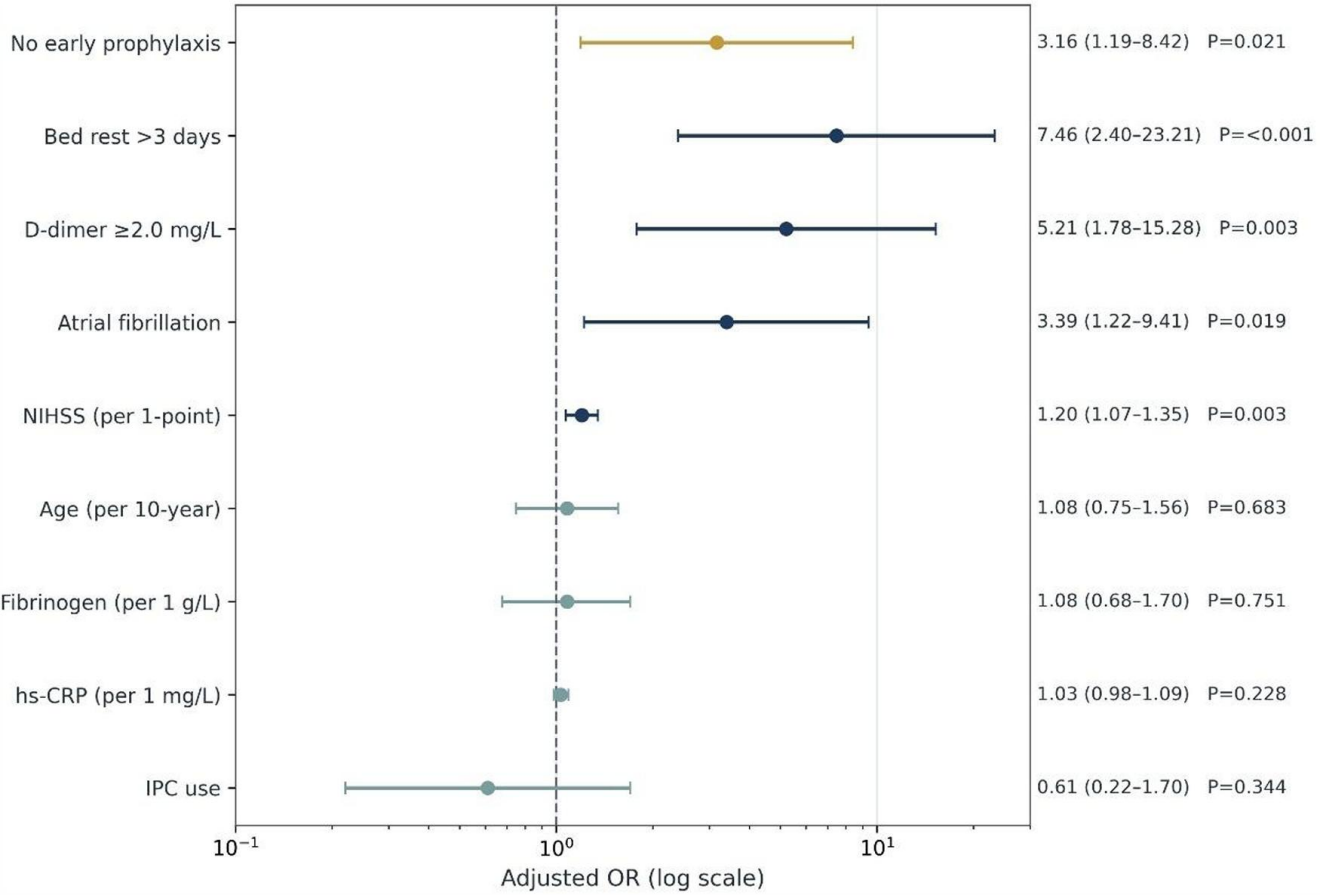
Forest plot of adjusted odds ratios for in-hospital DVT.

As shown in Table 2 (Model B), absence of early pharmacological thromboprophylaxis was associated with higher odds of in-hospital DVT after adjustment for measured covariates (adjusted OR = 3.16, 95% CI: 1.19–8.42, *P* = 0.021). Other independent predictors of DVT included prolonged absolute bed rest >3 days (OR = 7.46, 95% CI: 2.40–23.21, *P* < 0.001), D-dimer ≥2.0 mg/L (OR = 5.21, 95% CI: 1.78–15.28, *P* = 0.003), atrial fibrillation (OR = 3.39, 95% CI: 1.22–9.41, *P* = 0.019), and higher pre-thrombolysis NIHSS score (OR = 1.20 per point, 95% CI: 1.07–1.35, *P* = 0.003). Model calibration was acceptable according to the Hosmer-Lemeshow test (*P* = 0.672). The main adjusted effect estimates are visualized in Figure 2. Interpretation of this association should take into account the non-uniform DVT ascertainment strategy during hospitalization, as 59.4% of DVT events were diagnosed after symptom-triggered ultrasonography and 40.6% through screening examinations.

### 3.5 Secondary and supportive analyses

#### 3.5.1 Bleeding safety outcome

**Figure 3:**
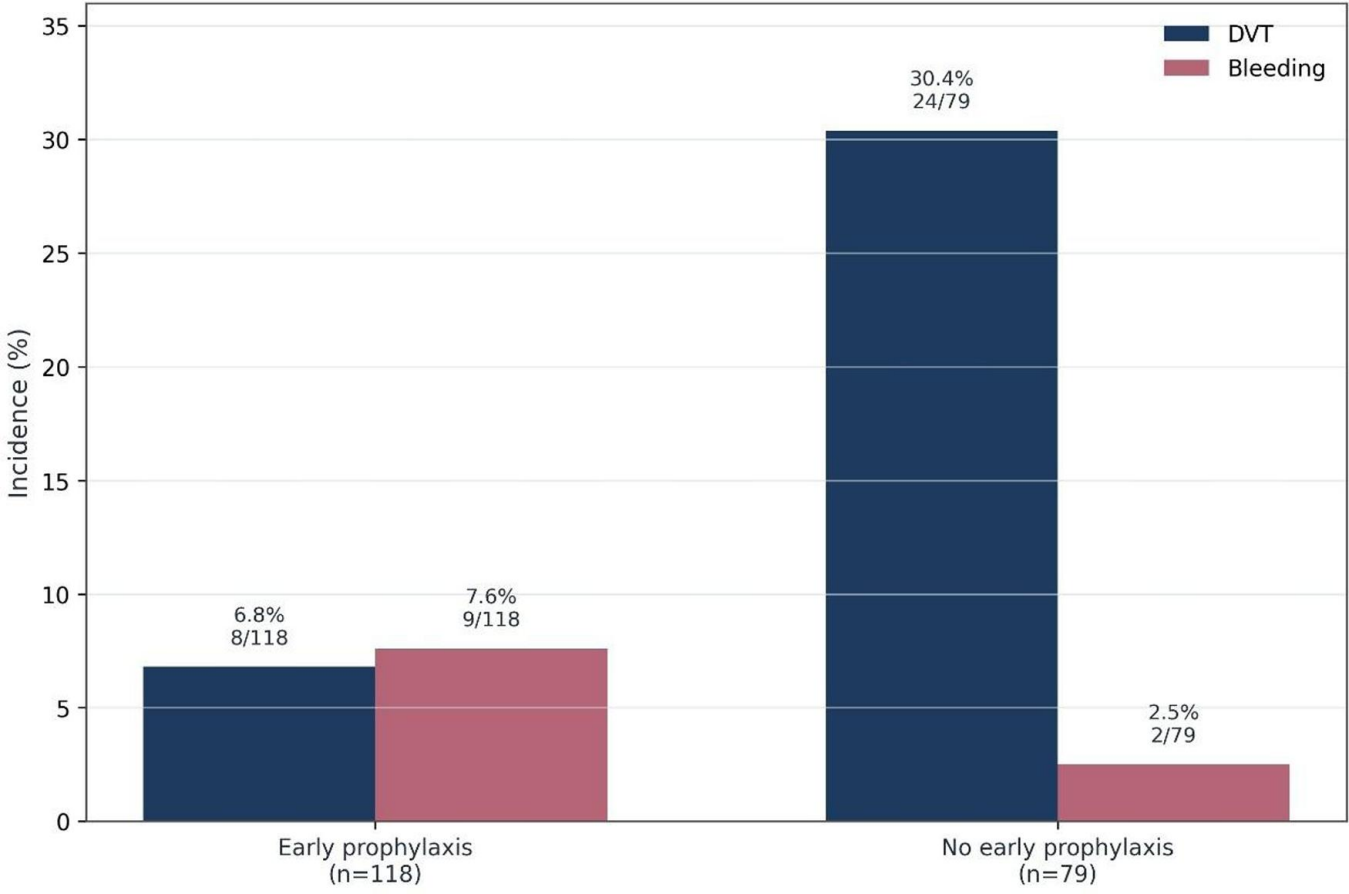
In-hospital DVT and bleeding outcomes by prophylaxis group.

Figure 3 compares DVT and bleeding outcomes between groups. Although bleeding events were numerically more frequent in the early prophylaxis group than in the no-early-prophylaxis group (7.6% vs. 2.5%), this difference was not statistically significant (crude OR = 3.16, 95% CI: 0.66–15.06, *P* = 0.148). After adjustment for age, NIHSS, and atrial fibrillation, the association remained non-significant (adjusted OR = 2.85, 95% CI: 0.57–14.23, *P* = 0.202). No fatal bleeding occurred during hospitalization. Detailed case-level information on bleeding events is presented in Supplementary Table S2.

#### 3.5.2 Subgroup analysis

Exploratory subgroup analyses showed a generally consistent direction of association across strata; however, these estimates were imprecise and should be interpreted cautiously because of limited sample size and event counts (Supplementary Table S3 and Supplementary Figure S1).

#### 3.5.3 Cumulative effect of high-risk features

**Figure 4:**
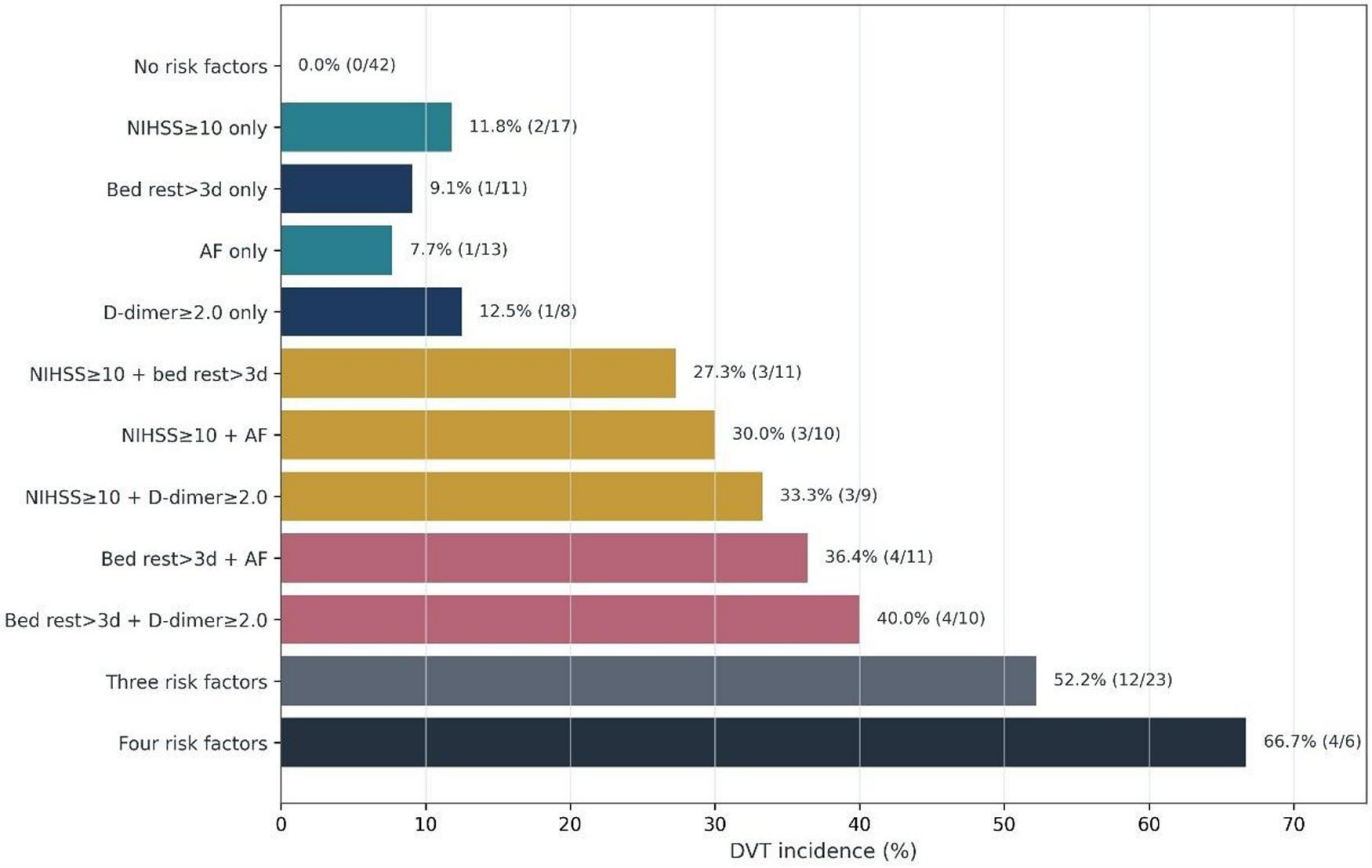
Detailed stratification of DVT incidence by cumulative risk factors.

Based on the independent predictors identified in Model B, four high-risk features were defined: NIHSS ≥10, bed rest >3 days, atrial fibrillation, and D-dimer ≥2.0 mg/L. As shown in Figure 4, DVT incidence increased progressively with the accumulation of these risk factors, rising from 2.6% (2/78) in patients with 0–1 features to 16.7% (10/60) in those with 2 features and 50.8% (20/59) in those with 3–4 features (*P* for trend < 0.001).

#### 3.5.4 Characteristics of in-hospital DVT events

Detailed clinical characteristics of the 32 patients who developed in-hospital DVT are summarized in Supplementary Table S4. Most DVT events involved the left lower limb (53.1%), and proximal DVT accounted for 65.6% of all cases. The median time from thrombolysis to DVT diagnosis was 5 days (IQR, 3–8), with most events diagnosed within 7 days of IVT. Symptom-triggered ultrasonography accounted for 59.4% of diagnoses, whereas 40.6% were detected on screening ultrasound. Four patients (12.5%) had confirmed pulmonary embolism, and one patient (3.1%) died of DVT-related causes.

Among patients who received early pharmacological thromboprophylaxis, prophylactic anticoagulation was continued for the duration of hospitalization (median 10 days, IQR 7–15) or until ambulation was restored. No patient was discharged on prophylactic anticoagulation unless already indicated for atrial fibrillation or prior VTE. Post-discharge follow-up for VTE events was not systematically performed, as this was an in-hospital outcome study. However, review of hospital readmission records within 90 days identified no additional cases of symptomatic pulmonary embolism or DVT-related rehospitalization among the 197 patients.

### 3.6 Sensitivity analyses

The main association remained directionally consistent in three sensitivity analyses, including modeling D-dimer as a log-transformed continuous variable, excluding patients with extremely short or long hospital stays, and additional adjustment for the propensity score (Supplementary Table S5).

## 4 Discussion

In this retrospective cohort of patients with acute ischemic stroke treated with intravenous thrombolysis, the absence of early pharmacological thromboprophylaxis was associated with a higher in-hospital DVT rate and remained statistically associated with DVT after adjustment for measured covariates. However, patients who did not receive early prophylaxis had substantially greater baseline severity, including older age, higher NIHSS scores, more frequent atrial fibrillation, more prolonged bed rest, and higher D-dimer levels, all well-established predictors of post-stroke DVT. Consequently, despite multivariable adjustment, residual confounding related to stroke severity, hypercoagulable state, and unmeasured clinician judgment (e.g., perceived bleeding risk) cannot be excluded. Therefore, our findings should be interpreted only as an association observed in routine practice and not as evidence of a causal effect of withholding prophylaxis itself. The observed odds ratio of 3.16 (95% CI: 1.19–8.42) may still be influenced by these underlying imbalances.

An additional consideration is outcome ascertainment. Lower extremity ultrasonography was not performed under a uniform surveillance protocol, but was partly symptom-triggered and partly based on screening of clinically perceived high-risk patients. This introduces the possibility of detection bias, as patients with more severe neurological deficits, prolonged immobility, or more complex clinical courses may have been more likely both to undergo ultrasonography and to be diagnosed with DVT. Accordingly, the observed association between lack of early prophylaxis and DVT may have been amplified, at least in part, by differential detection.

The overall incidence of in-hospital DVT in our cohort was 16.2%, which is consistent with previous domestic and international reports (14). More importantly, the multivariable analysis identified five independent factors associated with DVT: prolonged absolute bed rest (>3 days), higher pre-thrombolysis NIHSS score (per 1-point increase), D-dimer ≥2.0 mg/L, atrial fibrillation, and—crucially—absence of early pharmacological prophylaxis. These factors align with the classical Virchow's triad (venous stasis, endothelial injury, and hypercoagulability) and have been well documented in the literature (17–21).

Prolonged bed rest, reflecting venous stasis, was the strongest predictor (OR = 7.46). This finding underscores the importance of early mobilization and mechanical preventive measures, such as intermittent pneumatic compression (IPC), in immobilized patients (17, 18). The strong association between NIHSS score and DVT (OR = 1.20 per point) is consistent with previous studies showing that stroke severity is a key determinant of post-stroke complications, including DVT (16, 17). Severe neurological deficits limit mobility, providing a hemodynamic basis for thrombus formation.

D-dimer ≥2.0 mg/L, a marker of hypercoagulability and fibrin turnover, emerged as another powerful predictor (OR = 5.21). Elevated D-dimer not only reflects thrombotic burden from the index stroke but may also signal ongoing susceptibility to new thrombus formation, particularly in the setting of systemic inflammation and endothelial activation (19, 21). Atrial fibrillation (OR = 3.39) is well recognized as a source of cardioembolism, but our data demonstrate that it also confers a systemic pro-thrombotic milieu that increases the risk of venous thrombosis, consistent with previous reports (21, 22).

The observed association between lack of early prophylaxis and DVT is clinically relevant because it may reflect a vulnerable subgroup in whom thrombotic risk, neurological severity, and concern about bleeding converge. However, given the retrospective design and non-random treatment allocation, the present study cannot fully disentangle whether the excess DVT risk was attributable to omission of prophylaxis itself or to the underlying clinical severity that also influenced prophylaxis decisions (11, 13, 16).

To understand why some patients miss timely prophylaxis, we constructed a separate multivariable model (Model A) with “absence of early prophylaxis” as the outcome. Higher NIHSS, atrial fibrillation, and elevated D-dimer were independent predictors of not receiving prophylaxis, while age ≥75 years showed a borderline association. This pattern clearly illustrates a risk-treatment paradox: patients with the highest baseline thrombotic risk were paradoxically less likely to receive guideline-recommended early prophylaxis. The most likely explanation is clinician concern about post-thrombolysis hemorrhagic transformation. Specifically: (1) higher NIHSS often implies larger infarct volume, a well-established risk factor for symptomatic intracranial hemorrhage (sICH); (2) atrial fibrillation may raise concerns about cardioembolic stroke with larger core or microbleeds, and concomitant antiplatelet use is common; (3) elevated D-dimer, although a marker of hypercoagulability and DVT risk, may be misinterpreted as indicating active fibrinolysis or coagulopathy, leading to therapeutic caution. Additional unmeasured factors include: prior history of bleeding, labile blood pressure, renal impairment (affecting LMWH clearance), and physician experience or departmental protocols. Paradoxically, these clinical features — severe stroke, atrial fibrillation, elevated D-dimer — are precisely the ones that also predict DVT (as shown in Model B), creating a clinical dilemma where the highest-risk patients are least likely to receive the intervention that might reduce that risk. This paradox is not a criticism of individual clinicians but rather a signal that current post-thrombolysis prophylaxis decision-making lacks structured, protocol-driven support. Our findings highlight the need for a more structured, protocol-driven approach to post-thrombolysis prophylaxis that explicitly addresses this risk-benefit balance.

An essential aspect of any study examining thromboprophylaxis is the assessment of bleeding risk. In our cohort, bleeding events were numerically more frequent in the early prophylaxis group (7.6% vs. 2.5%), but the difference did not reach statistical significance (*P* = 0.123). After adjusting for potential confounders, the association remained non-significant (adjusted OR = 2.85, 95% CI 0.57–14.23). Importantly, no fatal bleeding occurred, and the majority of events were manageable with supportive care. These safety data, while limited by the small number of events, are reassuring and consistent with previous studies that have demonstrated the safety of LMWH prophylaxis when initiated after exclusion of intracranial hemorrhage (14, 15). Nevertheless, the narrow confidence interval indicates that we cannot exclude a modest increase in bleeding risk, and the decision to initiate prophylaxis must always be individualized, weighing the substantial DVT risk against the potential for bleeding.

Our findings extend those of previous investigations in several ways. Han et al. (16) developed a nomogram for DVT prediction in acute stroke patients after endovascular thrombectomy, identifying similar risk factors (NIHSS, bed rest, D-dimer). However, their study did not specifically examine the role of early pharmacological prophylaxis. Wang et al. (11) also constructed a nomogram for DVT in thrombolysed AIS patients, but again focused on risk prediction rather than preventive practices. Tøndel et al. (15), in a systematic review, emphasized the importance of early prophylaxis but noted that evidence in the IVT population is sparse. Our study directly addresses this gap by demonstrating that the prevention gap is independently associated with DVT, thereby supporting the need for prospective studies and standardized post-thrombolysis care pathways to clarify whether more consistent prophylaxis and surveillance practices may improve outcomes.

Babić et al. (22) and Barakzie et al. (21) highlighted the role of coagulation biomarkers, including D-dimer, in stroke risk stratification. Our results support the use of D-dimer not only as a prognostic marker but also as a tool to identify patients who might benefit most from early prophylaxis. The cumulative risk analysis (Figure 4) further illustrates that the DVT risk increases steeply with the number of high-risk features (NIHSS ≥10, bed rest >3 days, atrial fibrillation, D-dimer ≥2.0 mg/L), from 2.6% in those with 0–1 features to 50.8% in those with 3–4 features. This simple stratification could aid clinicians in prioritizing prophylaxis for the highest-risk patients.

**Figure 5:**
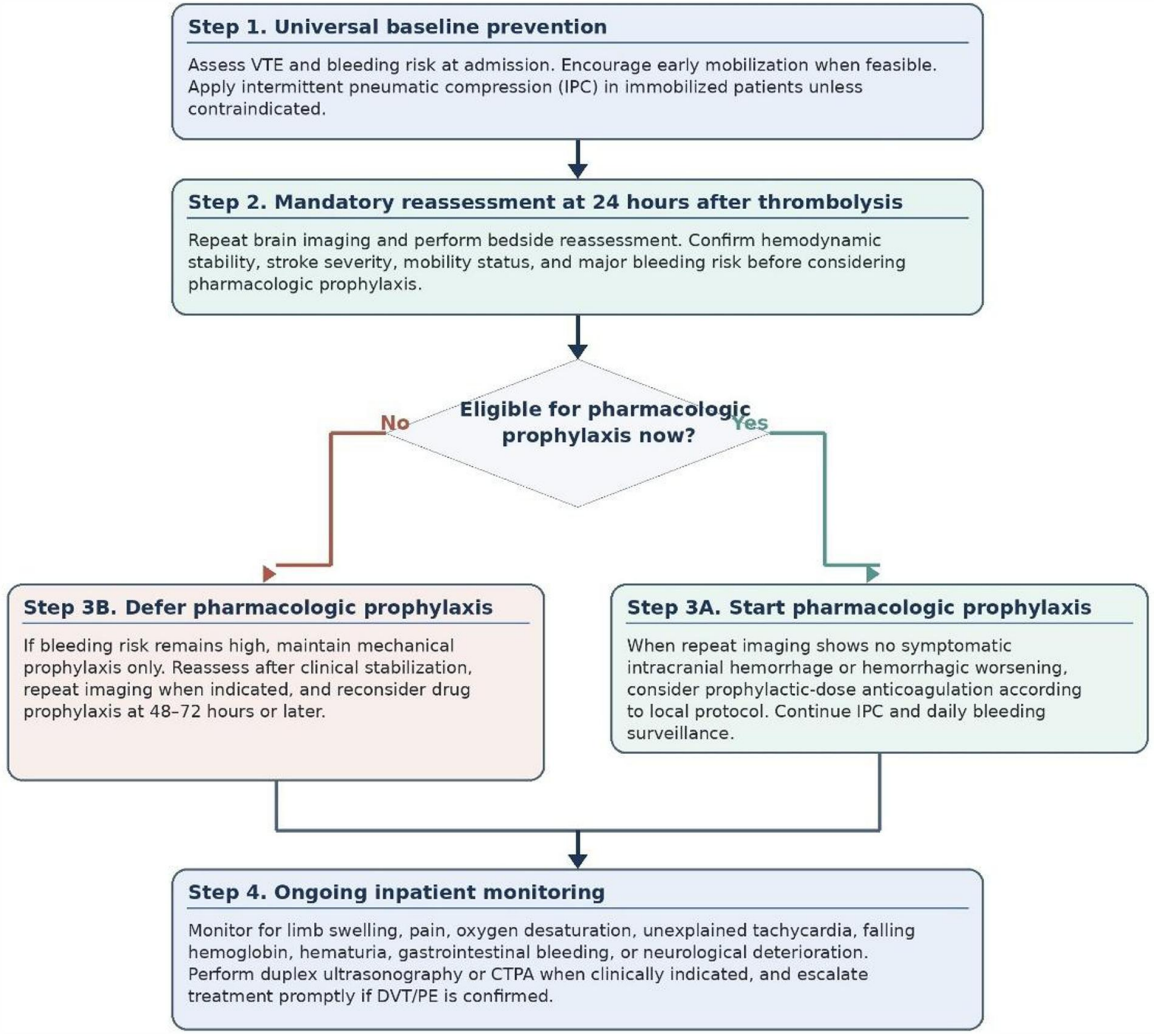
Proposed post-thrombolysis VTE prophylaxis clinical workflow.

Based on the present findings, we proposed a practical post-thrombolysis VTE prophylaxis workflow to assist risk stratification and bedside decision-making (Figure 5).

### 4.1 Clinical implications

Our study has several practical implications. First, it underscores the need for systematic risk assessment immediately after thrombolysis. Patients with severe neurological deficits, prolonged expected bed rest, atrial fibrillation, or markedly elevated D-dimer should be flagged as high priority for early prophylaxis. Second, the findings support the implementation of standardized post-thrombolysis care pathways that include a clear timeline for prophylaxis initiation. Such pathways should mandate repeat brain imaging at 24 h to exclude hemorrhage, followed by prompt initiation of pharmacological prophylaxis if safe. Third, our data highlight the importance of documenting and addressing the prevention gap as a quality metric in stroke care. Institutions could monitor the proportion of eligible patients who receive timely prophylaxis and use this as a benchmark for improvement.

Based on our findings and current guidelines, we propose an integrated management pathway (Supplementary Figure S1) that includes: (1) basic prophylaxis (education, ankle pumps, passive mobilization) for all patients immediately after admission; (2) mechanical prophylaxis (IPC) within 24 h unless contraindicated; (3) repeat brain imaging at 24 h; (4) initiation of pharmacological prophylaxis (LMWH or fondaparinux) 24–48 h post-thrombolysis if no hemorrhage is detected and bleeding risk is deemed acceptable; and (5) enhanced surveillance (e.g., periodic lower extremity ultrasound) for patients with multiple high-risk features.

### 4.2 Limitations

This study has several limitations that should be acknowledged. First, its single-center, retrospective design may introduce selection bias and information bias, despite our efforts to collect data systematically. Second, the sample size, particularly the limited number of DVT events (*n* = 32), restricts model stability and the precision of adjusted estimates. Although no significant multicollinearity was detected among covariates (all VIFs <2.5), the modest event count increases the risk of overfitting and widens confidence intervals. This limitation precludes more detailed subgroup analyses or examination of rarer outcomes such as pulmonary embolism. Therefore, our findings should be viewed as hypothesis-generating rather than definitive evidence of a causal protective effect of early thromboprophylaxis. Third, the definition of “early prophylaxis” (24–48 h) was based on clinical practice and guidelines, but the exact timing within this window could influence the risk-benefit balance; we did not have sufficient granularity to analyze prophylaxis timing as a continuous variable. Fourth, the decision to initiate early prophylaxis was not randomized but based on clinical judgment. Patients who did not receive prophylaxis had markedly higher baseline DVT risk (older age, higher NIHSS, atrial fibrillation, prolonged bed rest, elevated D-dimer). These factors are strong DVT predictors independent of prophylaxis. Hence, despite multivariable adjustment, residual confounding (e.g., infarct volume, clinician's bleeding risk assessment) and confounding by indication are highly probable: clinicians may have withheld prophylaxis from those already perceived as highest risk. Consequently, the adjusted OR of 3.16 may be inflated by these imbalances and should not be interpreted as a pure treatment effect. Fifth, the lack of a standardized DVT surveillance protocol introduces major detection bias. Ultrasonography was performed symptom-triggered or as screening in high-risk patients, not on a fixed schedule. Patients in the no-prophylaxis group—who had more severe strokes and longer immobilization—were therefore more likely to undergo ultrasound, leading to higher DVT detection independent of true prophylaxis effect. Thus, the observed association (OR 3.16) may be substantially inflated by detection bias. A prospective study with uniform ultrasound surveillance (e.g., day 5 for all patients) is needed to eliminate this bias. Sixth, we did not systematically collect data on the use of graduated compression stockings or other mechanical methods, which could have influenced outcomes. Moreover, our retrospective design could not capture individual clinical reasons for withholding prophylaxis, the proposed drivers (e.g., fear of hemorrhage, antiplatelet use) remain inferential; prospective studies with structured documentation are needed to confirm the risk-treatment paradox's underlying causes. Finally, the study focused on in-hospital DVT and did not capture post-discharge events, which may underestimate the total DVT burden.

The extreme baseline imbalance between groups raises concerns about propensity by severity. Although we adjusted for measured confounders (NIHSS, D-dimer, atrial fibrillation), important unmeasured or partially measured confounders may remain, including infarct size/location, physician's subjective threshold for anticoagulation, dynamic post-thrombolysis coagulation changes, and antiplatelet use. Additionally, the lack of a uniform DVT surveillance protocol meant that patients with more severe strokes or prolonged immobilization were more likely to undergo ultrasonography, potentially biasing the association toward a stronger effect. Therefore, while our findings identify a clinically important prevention gap, they should be viewed as hypothesis-generating and not sufficient to mandate universal early prophylaxis without prospective validation.

### 4.3 Future directions

Larger, multicenter prospective studies are warranted to validate our findings and to establish the optimal timing and regimen of pharmacological prophylaxis in this population. Randomized controlled trials comparing different prophylaxis strategies (e.g., early vs. delayed initiation, LMWH vs. fondaparinux, with or without IPC) would provide the highest level of evidence. Additionally, future research should explore the use of dynamic biomarkers (e.g., serial D-dimer measurements) to guide individualized prophylaxis decisions. Implementation science studies examining the effectiveness of clinical pathways and quality improvement interventions in reducing the prevention gap would also be valuable.

## 5 Conclusion

In patients with acute ischemic stroke treated with intravenous thrombolysis, absence of early pharmacological thromboprophylaxis was associated with higher odds of in-hospital DVT in unadjusted and adjusted analyses. However, given the marked baseline imbalances between groups and the likelihood of residual confounding and confounding by indication, this association should not be interpreted as evidence of a causal protective effect of early prophylaxis. Instead, our findings highlight a clinically relevant prevention gap that warrants prospective investigation. Future studies with standardized prophylaxis protocols, uniform DVT surveillance, and randomized or rigorously matched designs are required to determine whether consistent early prophylaxis can reduce DVT risk without increasing bleeding complications.

## Data Availability

The raw data supporting the conclusions of this article will be made available by the authors, without undue reservation.
