# Supplementary material for: Risk factors and intervention strategies for lower extremity deep vein thrombosis after intravenous thrombolysis for acute ischemic stroke
Source: Front Cardiovasc Med. 2026 Jul 7;13:1832515. doi: 10.3389/fcvm.2026.1832515 (PMC13386421; doi:10.3389/fcvm.2026.1832515)
Supplement: Supplementary file 1 [file Datasheet1.docx]

**Supplementary Table S1. Variables included in the propensity score model and balance diagnostics**

| Variable | Early prophylaxis group (n=118) | No early prophylaxis group (n=79) | Standardized mean difference (SMD) | SMD after propensity score adjustment |
| --- | --- | --- | --- | --- |
| Demographics |  |  |  |  |
| Age, years, mean ± SD | 66.8 ± 12.1 | 72.5 ± 11.8 | 0.48 | 0.12 |
| Age ≥75 years, n (%) | 36 (30.5) | 39 (49.4) | 0.39 | 0.08 |
| Male sex, n (%) | 75 (63.6) | 47 (59.5) | 0.08 | 0.03 |
| Medical history |  |  |  |  |
| Hypertension, n (%) | 85 (72.0) | 57 (72.2) | 0.01 | 0.02 |
| Diabetes mellitus, n (%) | 47 (39.8) | 32 (40.5) | 0.01 | 0.01 |
| Atrial fibrillation, n (%) | 26 (22.0) | 37 (46.8) | 0.54 | 0.09 |
| Previous stroke, n (%) | 35 (29.7) | 26 (32.9) | 0.07 | 0.04 |
| Clinical characteristics |  |  |  |  |
| Baseline NIHSS score before thrombolysis, median (IQR) | 4 (2, 8) | 12 (7, 18) | 0.89 | 0.14 |
| NIHSS ≥10, n (%) | 21 (17.8) | 43 (54.4) | 0.82 | 0.11 |
| Lower limb paralysis, n (%) | 42 (35.6) | 48 (60.8) | 0.52 | 0.10 |
| Complete bed rest >3 days, n (%) | 25 (21.2) | 40 (50.6) | 0.64 | 0.13 |
| Use of IPC, n (%) | 58 (49.2) | 24 (30.4) | 0.39 | 0.07 |
| Laboratory variables |  |  |  |  |
| D-dimer, mg/L, mean ± SD | 0.7 ± 0.6 | 1.5 ± 1.4 | 0.75 | 0.15 |
| D-dimer ≥2.0 mg/L, n (%) | 8 (6.8) | 28 (35.4) | 0.76 | 0.12 |
| Fibrinogen, g/L, mean ± SD | 3.4 ± 1.0 | 3.9 ± 1.2 | 0.45 | 0.08 |
| hs-CRP, mg/L, median (IQR) | 4.2 (1.9, 9.5) | 8.1 (3.5, 16.2) | 0.51 | 0.09 |
| Propensity score, mean ± SD | 0.32 ± 0.15 | 0.58 ± 0.21 | 1.42 | 0.06 |

**Supplementary Table S2. Detailed listing of bleeding events**

| **Patient No.** | **Group** | **Type of bleeding event** | **Time of occurrence (days after thrombolysis)** | **Severity** | **Management** | **Outcome** |
| --- | --- | --- | --- | --- | --- | --- |
| 1 | Early prophylaxis group | Symptomatic intracranial hemorrhage | 2 | Severe (NIHSS +4) | Discontinuation of anticoagulation; conservative treatment | Recovered |
| 2 | Early prophylaxis group | Symptomatic intracranial hemorrhage | 3 | Severe (NIHSS +6) | Discontinuation of anticoagulation; conservative treatment | Moderate disability |
| 3 | Early prophylaxis group | Symptomatic intracranial hemorrhage | 4 | Severe (NIHSS +5) | Discontinuation of anticoagulation; conservative treatment | Recovered |
| 4 | No early prophylaxis group | Symptomatic intracranial hemorrhage | 5 | Severe (NIHSS +8) | Conservative treatment | Severe disability |
| 5 | Early prophylaxis group | Major gastrointestinal bleeding | 3 | Severe (Hb decrease of 3.2 g/dL) | Blood transfusion; discontinuation of anticoagulation | Recovered |
| 6 | Early prophylaxis group | Major gastrointestinal bleeding | 4 | Moderate (Hb decrease of 2.1 g/dL) | Discontinuation of anticoagulation | Recovered |
| 7 | Early prophylaxis group | Major gastrointestinal bleeding | 6 | Moderate (Hb decrease of 1.8 g/dL) | Temporary interruption of anticoagulation followed by resumption | Recovered |
| 8 | Early prophylaxis group | Hematuria (major bleeding) | 5 | Moderate (Hb decrease of 2.5 g/dL) | Temporary interruption of anticoagulation | Recovered |
| 9 | Early prophylaxis group | Major puncture-site hematoma | 2 | Moderate | Compression; anticoagulation continued | Recovered |
| 10 | Early prophylaxis group | Retroperitoneal hematoma | 7 | Severe (Hb decrease of 4.1 g/dL) | Blood transfusion; discontinuation of anticoagulation | Recovered |
| 11 | No early prophylaxis group | Gastrointestinal bleeding (non-major) | 8 | Mild | Observation | Recovered |

**Supplementary Table S3. Subgroup analysis of the association between absence of early prophylaxis and in‑hospital DVT**

| Subgroup | Early prophylaxis (n=118) | No early prophylaxis (n=79) | Absolute risk increase | Crude OR (95% CI) | *P* value |
| --- | --- | --- | --- | --- | --- |
| NIHSS <10 | 2/97 (2.1%) | 7/36 (19.4%) | 17.30% | 11.5 (2.3–58.5) | 0.003 |
| NIHSS ≥10 | 6/21 (28.6%) | 17/43 (39.5%) | 10.90% | 1.64 (0.52–5.17) | 0.394 |
| Bed rest ≤3 days | 2/93 (2.2%) | 5/39 (12.8%) | 10.60% | 6.68 (1.24–36.1) | 0.027 |
| Bed rest >3 days | 6/25 (24.0%) | 19/40 (47.5%) | 23.50% | 2.86 (0.94–8.69) | 0.061 |
| Atrial fibrillation (-) | 4/92 (4.3%) | 10/42 (23.8%) | 19.50% | 6.88 (2.02–23.4) | 0.002 |
| Atrial fibrillation (+) | 4/26 (15.4%) | 14/37 (37.8%) | 22.40% | 3.36 (0.96–11.8) | 0.056 |
| D‑dimer <2.0 mg/L | 5/110 (4.5%) | 9/51 (17.6%) | 13.10% | 4.50 (1.43–14.1) | 0.01 |
| D‑dimer ≥2.0 mg/L | 3/8 (37.5%) | 15/28 (53.6%) | 16.10% | 1.92 (0.39–9.49) | 0.424 |

**Supplementary Table S4. Detailed characteristics of patients with in-hospital DVT (n = 32)**

| Variable | Value |
| --- | --- |
| DVT location, n (%) |  |
| - Left lower extremity | 17 (53.1) |
| - Right lower extremity | 10 (31.3) |
| - Bilateral | 5 (15.6) |
| DVT type, n (%) |  |
| - Proximal DVT (popliteal vein or above) | 21 (65.6) |
| - Distal DVT (calf intramuscular veins) | 11 (34.4) |
| Time from thrombolysis to DVT diagnosis, days, median (IQR) | 5 (3–8) |
| Distribution of time to DVT diagnosis, n (%) |  |
| - ≤3 days | 8 (25.0) |
| - 4–7 days | 15 (46.9) |
| - 8–14 days | 7 (21.9) |
| - >14 days | 2 (6.3) |
| Method of DVT diagnosis, n (%) |  |
| - Symptom-triggered ultrasonography (limb swelling/pain) | 19 (59.4) |
| - Routine screening ultrasonography | 13 (40.6) |
| Management after DVT diagnosis, n (%) |  |
| - Initiation of therapeutic-dose anticoagulation | 24 (75.0) |
| - Continued prophylactic-dose anticoagulation | 5 (15.6) |
| - Mechanical prophylaxis only | 3 (9.4) |
| Concomitant PE (confirmed), n (%) | 4 (12.5) |
| DVT-related mortality, n (%) | 1 (3.1) |

**Supplementary Table S5. Sensitivity analyses for the association between the absence of early prophylaxis and in-hospital DVT**

| **Sensitivity analysis** | **Adjusted OR (95% CI)** | **P value** |
| --- | --- | --- |
| Primary analysis (original model) | 3.16 (1.19–8.42) | 0.021 |
| Analysis 1: D-dimer modeled as a continuous variable (log-transformed) | 3.02 (1.13–8.07) | 0.027 |
| Analysis 2: Excluding patients with hospital stay <3 days or >30 days (n = 24) | 3.54 (1.25–10.03) | 0.017 |
| Analysis 3: Propensity score-adjusted model | 2.98 (1.08–8.22) | 0.035 |
| Analysis 4: NIHSS modeled as a categorical variable (<5, 5–9, ≥10) | 3.21 (1.20–8.58) | 0.020 |
| Analysis 5: D-dimer modeled as a categorical variable (<1.0, 1.0–1.9, ≥2.0 mg/L) | 3.08 (1.15–8.26) | 0.025 |
| Analysis 6: Multilevel model with admission year as a random intercept | 3.11 (1.16–8.34) | 0.024 |
| Analysis 7: E-value analysis | E-value = 5.72 | — |


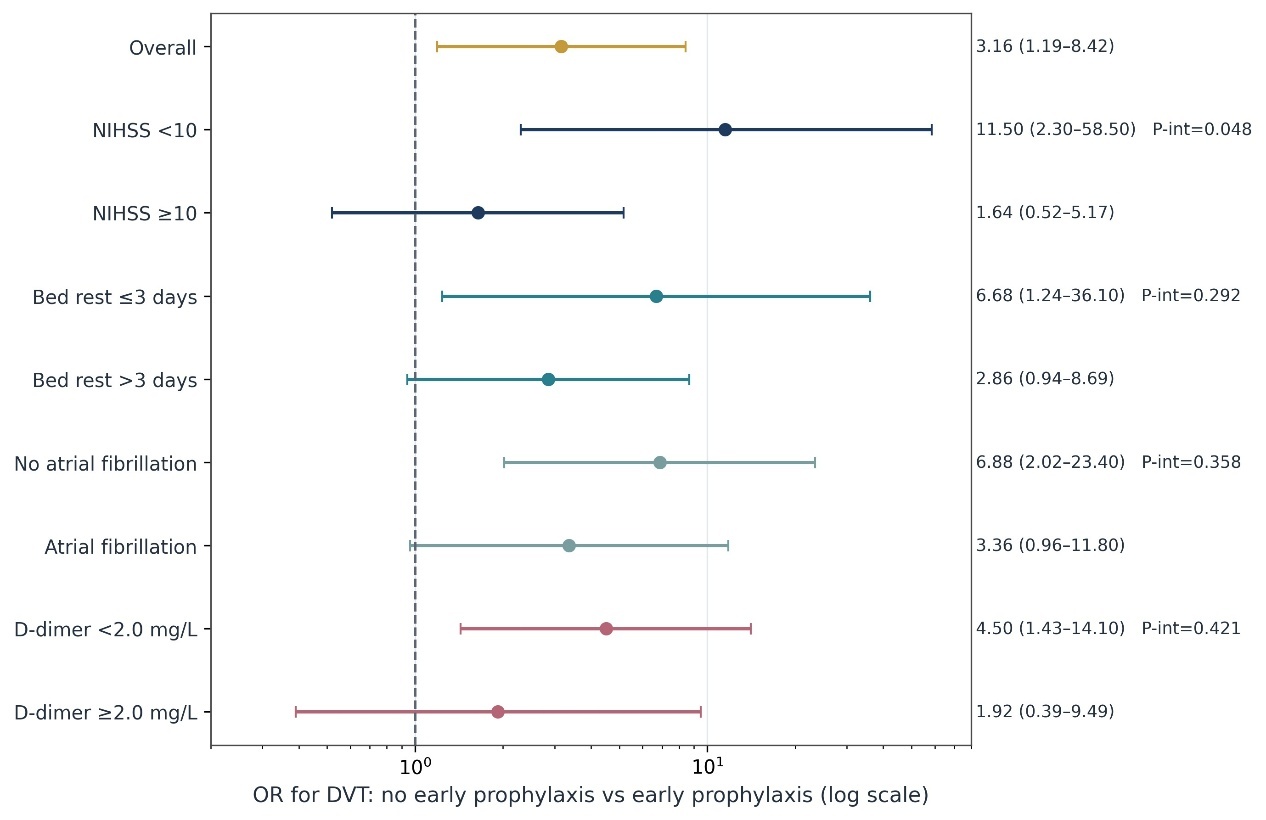


**Supple Figure 1. Subgroup analysis: effect of early prophylaxis on DVT prevention across patient subgroups**
